# Human tolerogenic dendritic cell subtypes exert divergent effects on induction of cytotoxic CD4^+^ T cells

**DOI:** 10.3389/fimmu.2025.1698413

**Published:** 2025-12-22

**Authors:** Gabrielle Barran, Najib Naamane, Abdul Mannan Baru, Amy E. Anderson, Jane Falconer, Catharien M. U. Hilkens

**Affiliations:** 1 Translational and Clinical Research Institute, Newcastle University, Newcastle upon Tyne, United Kingdom; 2 John Dawson Drug Discovery and Development Institute, University of Sunderland, Sunderland, United Kingdom; 3 Immunology Network, Immunology Research Unit, GlaxoSmithKline, Stevenage, United Kingdom

**Keywords:** autoimmune diseases, cytotoxic CD4+ T cells, dexamethasone, rheumatoid arthritis, tolerogenic dendritic cells, vitamin D3

## Abstract

Tolerogenic dendritic cells (tolDC) are currently in clinical trials for the treatment of autoimmune diseases such as rheumatoid arthritis and multiple sclerosis. The methods for producing therapeutic tolDC vary widely, with little being known about the commonalities and differences of distinct cell products in terms of their regulatory actions on CD4^+^ T cells. We compared human monocyte-derived tolDC generated with vitamin D3 alone or in combination with dexamethasone. We found marked differences in the surface expression of HLA-DR and immune regulatory molecules, but also found commonalities, e.g. a strongly reduced capacity to produce interleukin-12 and a concomitant decreased ability to induce interferon-γ secretion by allogeneic CD4^+^ T cells. To gain a deeper understanding of how these tolDC types exert their regulatory effects, we co-cultured them with CD4^+^ T cells from rheumatoid arthritis patients or healthy controls and analysed the gene expression profile and function of the responding T cells. We found that tolDC generated with vitamin D3 alone, but not in combination with dexamethasone, induced potent cytotoxic activity in the responding CD4^+^ T cells as demonstrated by an enhanced cytotoxic gene signature, increased levels of intracellular granzyme B, and superior cytotoxic activity towards myeloid and B cells. These data identify cytotoxicity as an atypical CD4^+^ T helper cell effector function induced by some but not all tolDC types, with implications for their individual clinical applications.

## Introduction

Autoimmune diseases such as rheumatoid arthritis (RA) and multiple sclerosis (MS) are debilitating conditions, caused by a breach in immune tolerance to self-tissues and leading to uncontrolled and pathogenic immune responses to auto-antigens. Current therapeutics mainly act through dampening inflammation, but do not target the underlying cause of these pathologies. To counter this, tolerogenic dendritic cell (tolDC) therapies have been developed to reinstate immune tolerance in an antigen-specific manner, and a number of these have been or are being tested in clinical trials (1, 2).

Human tolDC are commonly generated from peripheral blood monocytes and a variety of methods have been described to endow these cells with stable tolerogenic function (3). These include the use of immunosuppressive agents at various stages of the monocyte-derived DC (moDC) differentiation process to attenuate their maturation into immunogenic cells. In general, tolDC are characterised by a ‘semi-mature’ phenotype expressing variable levels of MHC II, intermediate expression of co-stimulatory CD86, increased expression of regulatory molecules (e.g., PD-L1/2, MerTK and ILT3), as well as an anti-inflammatory cytokine secretion profile (e.g., low IL-12p70 and high IL-10). However, despite these typical features, a common transcriptomic ‘signature’ amongst different tolDC types has not been found, making it likely that these cells act upon T cells through both overlapping and distinct pathways (4, 5).

Phase I clinical trials with a number of tolDC types in several autoimmune diseases (including RA, MS, type I diabetes and Crohn’s disease) have shown that this therapeutic approach is safe and well-tolerated by patients (6). However, although there is anecdotal evidence for some clinical improvements in some patients, unequivocal and long-lasting clinical benefits of this treatment have not yet been established. The current challenge for the field is, therefore, to improve the efficacy of tolDC treatment to meet its enormous theoretical potential. Although a number of promising initiatives are ongoing to achieve this, e.g. through clinical trials that compare different routes of tolDC administration (7), progress is hampered by an incomplete understanding of how these cells exert their regulatory effects. This is further compounded by the variety of methods utilised to generate tolDC and their application in different autoimmune disease settings; making comparisons between different tolDC types difficult.

Two of the most commonly used agents for the generation of tolDC are the glucocorticoid dexamethasone (Dex) and the active form of vitamin D_3_ (VD_3_). We have developed therapeutic tolDC for the treatment of RA, using a combination of these immunosuppressive agents (referred to as DexVD_3_DC). We have shown that these tolDC have potent regulatory activity *in vitro*, including the induction of IL-10-producing Tr1-like cells (8–12). We have also shown the therapeutic benefit of equivalent murine tolDC, generated with Dex and VD_3_, in experimental models of inflammatory arthritis (13, 14). Our phase 1 clinical trial (AuToDeCRA – Autologous Tolerogenic Dendritic Cells for RA) showed that injection of these autologous tolDC into an inflamed joint is safe (11), and we have recently completed recruitment of RA patients to a phase 2a clinical trial (AuToDeCRA-2) in which we are testing different routes of tolDC administration (intra-articular, intra-dermal and intra-nodal). DexVD_3_DC are also undergoing testing in the TOLERANT trial at University Medical Centre Utrecht (15).

Addition of VD_3_ (in the absence of Dex) during differentiation of human monocytes into DC is another common method for the generation of tolDC (VD_3_DC). These VD_3_DC have decreased expression of co-stimulatory molecules and IL-12p70, with reduced ability to induce T cell proliferation and prime Th1 responses (16, 17). VD_3_DC have also been shown to induce antigen-specific hyporesponsiveness in CD4^+^ T cells and to promote the induction of Tr1-like cells or FoxP3^+^ Tregs *in vitro* (18–20). Equivalent murine tolDC, generated with VD_3_ as the sole tolerogenic agent, suppressed disease symptoms in the experimental autoimmune encephalomyelitis model and improved the balance between regulatory and pathogenic T cells (21–24). VD_3_DC have been trialled in MS patients (7).

Here, we investigated the regulatory properties of human monocyte-derived DexVD_3_DC and VD_3_DC. We performed phenotypic profiling of these tolDC types and investigated their regulatory actions on CD4+ T cells. We performed transcriptomic analyses of CD4^+^ T cells from RA patients that had been primed by tolDC, as well as functional studies. Our data show overlapping but also divergent properties of these tolDC types, particularly regarding the induction of cytotoxic activity in CD4^+^ T cells.

## Materials and methods

The minimum information about tolerogenic antigen presenting cells (MITAP) checklist was used for this paper (25).

### Peripheral blood samples and ethical approval

Ethical approval for the use of leukocyte reduction system (LRS) cones from platelet donations to the National Health Service Blood and Transplant (NHSBT) was provided by the Faculty of Medical Sciences Ethics Committee. For work carried out at GlaxoSmithKline, research use was in accord with the terms of the informed consents under an IRB/EC approved protocol. Ethical approval for the use of peripheral blood donations obtained from healthy volunteers with informed consent was provided by The Animal Welfare and Ethical Review Body (AWERB), Newcastle University. RA blood samples were obtained with informed consent from the Northeast Early Arthritis Clinic (NEAC) (Musculoskeletal Unit, Freeman Hospital, Newcastle-upon-Tyne Hospital Trust). Ethical approval was given by the Newcastle & North Tyneside 2 Research Ethics Committee under the project titled ‘Prognostic and therapeutic biomarkers in an observational inception cohort: the Northeast Early Arthritis Cohort’ (REC reference 12/NE/0251). Stored PBMC samples from four treatment-naïve early arthritis female patients, age range 46-68, with a confirmed diagnosis of RA were used.

### Generation of monocyte-derived mature and tolerogenic DC subtypes

Peripheral blood mononuclear cells (PBMC) were isolated by density gradient centrifugation on Lymphoprep (StemCell Technologies, Vancouver, Canada). CD14^+^ monocytes were freshly isolated from PBMC by magnetic positive selection using anti-CD14 MicroBeads (Miltenyi Biotec, Bergisch Gladbach, Germany), as previously described in detail (26). Monocytes were seeded at 0.5x10^6^ cells/ml in a 24-well plate (1ml/well) in RF10; RPMI-1640 supplemented with 10% v/v foetal bovine serum (FBS) (Gibco, Waltham, MA, USA), 100U/ml penicillin, 100µg/ml streptomycin and 2mM L-glutamine (Sigma-Aldrich, Burlington, MA, USA). To generate moDC, cells were cultured in the presence of GM-CSF and IL-4 (each 50 ng/ml; Immunotools, Friesoythe, Germany) and incubated at 37 °C, 5% CO_2_ for 7 days. Medium supplemented with cytokines (50ng/ml) was refreshed on day 3. On day 6, cells were either stimulated for 24hrs with 100ng/ml Lipopolysaccharide (LPS) (Sigma) to generate mature (Mat)DC or were left untouched as immature (Im)DC. To generate DexVD_3_DC, dexamethasone (Dex) (Sigma) was additionally added at 1μM on days 3 and 6 of culture and 0.1nM 1,25-dihydroxyvitamin D3 (Calcitriol) (VD_3_) (Tocris, Bristol, UK) was added alongside Dex and LPS on day 6 of culture. VD_3_DC were generated as described above with the addition of 10nM VD_3_ on days 0 and 3 of culture and LPS on day 6. On culture day 7, DC were harvested and washed extensively for further processing (see below). Viability and cell number was determined by trypan blue exclusion (Sigma).

### T cell isolation

CD4^+^ T cells were isolated from LRS cones using the RosetteSep human CD4^+^ T cell enrichment cocktail (StemCell) according to manufacturer’s instruction with the exception of adding 75µl cocktail/ml of sample. The sample was diluted 1:2 with PBS supplemented with 2% FBS (Sigma) and CD4^+^ T cells were separated by density gradient centrifugation on Lymphoprep (StemCell). For further isolation of naïve CD4^+^ T cells, CD45RO^+^ cells were depleted using CD45RO MicroBeads (Miltenyi Biotec), according to manufacturer’s instruction. Purity of naïve CD4^+^ T cells was confirmed by flow cytometry and was typically >85% (data not shown). Cells were cryopreserved using FBS (Sigma) supplemented with 10% dimethyl sulfoxide (DMSO) (Wak-Chemie Medical GmbH, Steinbach, Germany).

### Cell surface phenotyping of moDC

Cells were stained for viability using Zombie Aqua™ or Zombie NIR™ (Biolegend, San Diego, CA, USA) according to manufacturer’s instruction. The reaction was quenched with cold staining buffer (Ca^2+^ and Mg^2+^ free PBS supplemented with 3% FBS, 1mM EDTA and 0.01% sodium azide). To prevent non-specific antibody binding, cells were centrifuged and resuspended in staining buffer supplemented with 10% TruFcX Fc receptor block (Biolegend) for 20 minutes at 4 °C. Cells were stained for phenotypic analysis using the following mouse anti-human antibodies: CD83 - FITC (HB25e), CD86 - BV785 (IT2.2), HLA-DR - BV510 (L243), PD-L1 - PE-Cy7 (MIH1), MERTK - PE (590H11G1E) and ILT3 - APC (ZM4.1) in staining buffer supplemented with 10% Brilliant Buffer Plus and 5% True Stain Monocyte Blocker (Biolegend) for 30 minutes at 4°C. Cells were acquired immediately using the Attune NxT flow cytometer. UltraComp eBeads™ (Invitrogen) were used for compensation according to manufacturer’s instruction. Cells were protected from light throughout staining and acquisition. Data was analysed using FlowJo software version 10 (Treestar Inc, OR, USA). Fluorescence minus one (FMO) controls were used to gate live cells following exclusion of debris and doublets.

### Cytokine analysis

Concentrations of cytokines in culture supernatants were quantified using either the 18plex ProcartaPlex™ Human Th1/Th2/Th9/Th17 Cytokine Panel (Thermo) or the V-plex Plus Human Proinflammatory Panel 1 Kit (Meso Scale Discovery, Rockville, MD, USA) according to manufacturer’s instruction.

### MoDC/CD4^+^ T cell co-cultures

Cryopreserved CD4^+^ T cells were thawed, labelled with 0.5 µM CellTrace^™^ Far Red proliferation tracking dye (Invitrogen, Waltham, MA, USA) according to manufacturer’s instruction. Labelled CD4^+^ T cells were then co-cultured with freshly generated moDC (1x10^4^/well). Co-culture was carried out in 96-well U bottom plates at a DC: T cell ratio of 1:10 (1x10^5^ T cells/well) in RF10 at 37°C, 5% CO_2_ for 6 days. On day 6, supernatants were collected and stored at -80 °C. Cells were stained for viability as described above and surface stained for flow cytometry using mouse anti-human CD3 - BUV395 (UCHT1) and CD4 - PE-Cy7 (RPA-T4) in staining buffer supplemented with 4µg/ml human IgG (Hizentra^®^; CSL Behring, King of Prussia, PA, USA) to prevent non-specific antibody binding. Cells were incubated for 30 minutes at 4°C. Cells were centrifuged and resuspended in staining buffer to be acquired immediately on the BD LSR Fortessa X20^™^ or fixed in PBS supplemented with 1% formaldehyde. Fixed cells were stored at 4 °C protected from light and acquired within 7 days. BD^™^ CompBeads (BD Biosciences) were used for compensation according to manufacturer’s instruction. Data was analysed using FlowJo software version 10 (Treestar Inc, OR, USA).

### Gene expression profiling by Nanostring

Cryopreserved PBMC from early arthritis patients were thawed and labelled with CellTrace^™^ as above and co-cultured with freshly generated allogeneic healthy donor moDC at a 1:10 DC: PBMC ratio (1x10^5^ moDC and 1x10^6^ PBMC/well) in a 24-well plate in RF10 at 37°C, 5% CO_2._ On day 6, the cells were harvested and stained for fluorescence activated cell sorting (FACS) using viability dye Zombie NIR^™^ and the following mouse anti-human antibodies: CD3-BUV395 (UCHT1), CD4-PE-Cy7 (RPA-T4), CD11c-PE/Dazzle™ 594 (BU15) and CD14-FITC (M5E2) in staining buffer supplemented with human IgG (Hizentra^®^). Cells were strained through a 35μm cell strainer and acquired at 4 °C on the BD FACSAria^™^ II using a 70μm nozzle. Events were gated sequentially as follows: cells, single cells, live cells (Zombie NIR^-^), proliferated cells (CellTrace^-^), CD11c^-^, CD14^-^ and CD3^+^CD4^+^. Approximately 3 x 10^5^ cells were sorted into cold RF10 per sample. Sorted CD4^+^ T cells that had proliferated in response to matDC (T-MatDC), DexVD_3_DC (T-DexVD_3_DC) or VD_3_DC (T-VD_3_DC) were lysed in RLT buffer containing 1% β-mercaptoethanol, passed through a QIAshredder column (Qiagen, Hilden, Germany) and stored at -80°C. Total RNA was extracted using the RNeasy^®^ Micro Kit (Qiagen) according to manufacturer’s instruction. RNA concentration was determined using Qubit (Invitrogen) and 100ng RNA per sample was analysed on the NanoString nCounter^®^ platform using the Immune Exhaustion panel (NanoString Technologies, Seattle, WA, USA). Raw count data is provided in **Supplementary Data Sheet 1**.

### Analysis of gene expression data

Analysis of nCounter^®^ gene expression data was performed using R software version 4.2.1 in association with the Bioconductor repository (27). The NanoTube R package (28) was used for data processing and quality control (QC). Quality control of the gene expression count data was performed as recommended by NanoString^®^ Gene Expression Data Analysis Guidelines. In brief, the data was normalised in two steps using positive spike-in controls and geNorm-selected housekeeping genes (29). Removal of any outlier samples was performed based on different QC flags as recommended in NanoString^®^ Gene Expression Data Analysis Guidelines. Differential gene expression analysis was performed by fitting negative binomial generalised linear models to the raw count data using the DESeq2 Bioconductor package (30). To compare variation between groups, a likelihood ratio test (LRT) was performed, followed by Wald tests for pairwise comparisons between T-MatDC, T-DexVD_3_DC and T-VD_3_DC. Genes with a raw p-value < 0.05 and a Benjamini-Hochberg adjusted p-value < 0.05 for the LRT and Wald test, respectively, were considered to be differentially expressed. Gene Set Enrichment Analysis (GSEA) was conducted using the Fast Gene Set Enrichment Analysis (FGSEA) method outlined by Korotkevich et al. (31) which estimates gene set p-values based on the adaptive multi-level split Monte-Carlo approach. Gene sets were defined based on the NanoString Immune Exhaustion panel annotations and were considered significant if they had a p-value < 0.05. Principal Components Analysis (PCA) was performed using *stats::prcomp* R function. For hierarchical clustering, centred Pearson correlation and complete linkage were used as distance metric and agglomeration method, respectively. The results were visualised using *factoextra::fviz_pca*, *volcano3D::volcano3D* (32) and *NMF::aheatmap* R functions. Transcription factor (TF) activities were inferred using the Univariate Linear Model (ULM) method within the decoupleR package. Using the gene-level Wald statistics and the human CollecTRI regulatory network as input, UML regresses the TF-Gene interaction signed weights from the regulatory network against the differential expression statistics to compute activity scores per TF.

### Intracellular staining for granzyme B

Freshly generated moDC (1x10^4^/well) were co-cultured with thawed, allogeneic naïve T cells in 96-well U bottom plates at a 1:10 DC: T cell ratio in RF10 at 37 °C, 5% CO_2_ for 6 days. On day 6, cells were harvested, stained for viability as previously mentioned and fixed using Fixation/Permeabilisation Buffer (eBioscience) for 30 minutes at 4°C. Cells were permeabilised using Permeabilisation Buffer (eBioscience) for 15 minutes at 4°C. To block non-specific staining, cells were blocked with 2% mouse serum (Sigma) for 15 minutes prior to a 30-minute incubation with mouse anti-human granzyme B antibody - AF647 (GB11) at 4°C. Cells were washed, resuspended in staining buffer and acquired immediately on the BD FACSymphony™ A5. Data was analysed using FlowJo software version 10 (Treestar Inc, OR, USA).

### Cytotoxicity assay

T cells were primed in co culture with moDC for 6 days as mentioned above. On day 6, primed T cells were labelled with Tag-it Violet^™^ (Biolegend) to differentiate them from the target PBMC and 1x10^5^/well were co-cultured with thawed PBMC from the original moDC donor in 96-well U bottom plates at a 2:1 T cell: PBMC ratio in RF10 at 37 °C, 5% CO_2_ for 24 hours. After 24 hours, cells were stained with Zombie Aqua^™^ and Annexin V (Biolegend) for evaluation of apoptosis. To identify different cell populations, cells were stained with the following mouse anti-human antibodies: CD3 - BUV395 (UCHT1), CD19 - BV785 (HIB19) and CD11c - PE/Dazzle™ 594 (BU15) alongside human IgG (Hizentra^®^). Cells were washed and fixed in PBS supplemented with 1% formaldehyde. Fixed cells were stored at 4 °C protected from light and acquired on the BD FACSymphony™ A5 within 7 days. BD^™^ CompBeads (BD Biosciences) were used for compensation according to manufacturer’s instruction. Data was analysed using FlowJo software version 10 (Treestar Inc, OR, USA).

### Statistical analysis

Statistical analysis for flow cytometry and cytokine data was performed using Prism version 10 (GraphPad Software, San Diego, CA, USA). Statistical significance was determined by performing Kruskal-Wallis and Dunn’s multiple comparisons tests or a one-way ANOVA with Tukey’s paired *post hoc* analysis on ln-transformed data. Results were considered significant when p < 0.05.

## Results

### Phenotype and cytokine profile of DexVD_3_DC and VD_3_DC

**Figure 1:**
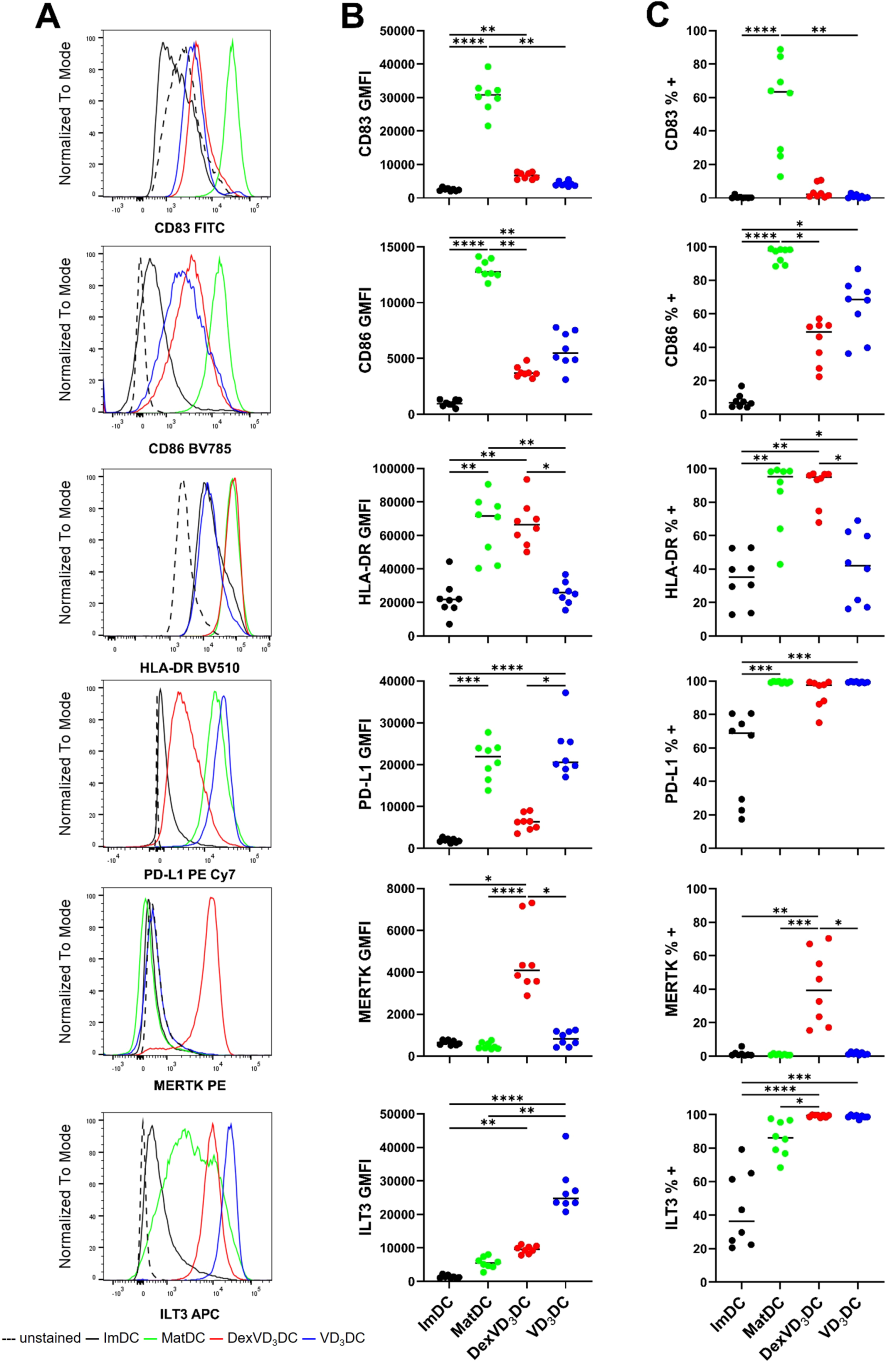
Phenotypic analysis of maturation and regulatory- associated markers in DexVD3DC and VD3DC compared to ImDC and MatDC controls. **(A)** Fluorescence Intensity, **(B)** Geometric Mean Fluorescence Intensity (GMFI), and **(C)** Percentage positive of CD83, CD86, HLA-DR, PD-L1, MERTK and ILT3, respectively, were measured using flow cytometry. Histograms **(A)** are representative of eight independent experiments. Data shown as individual values where n = 8 with a horizontal line representing the median value. Statistical significance was determined by performing Kruskal-Wallis and Dunn’s multiple comparisons tests. Significance is represented as *p < 0.05, **p < 0.01, ***p < 0.001 and ****p < 0.0001.

**Figure 2:**
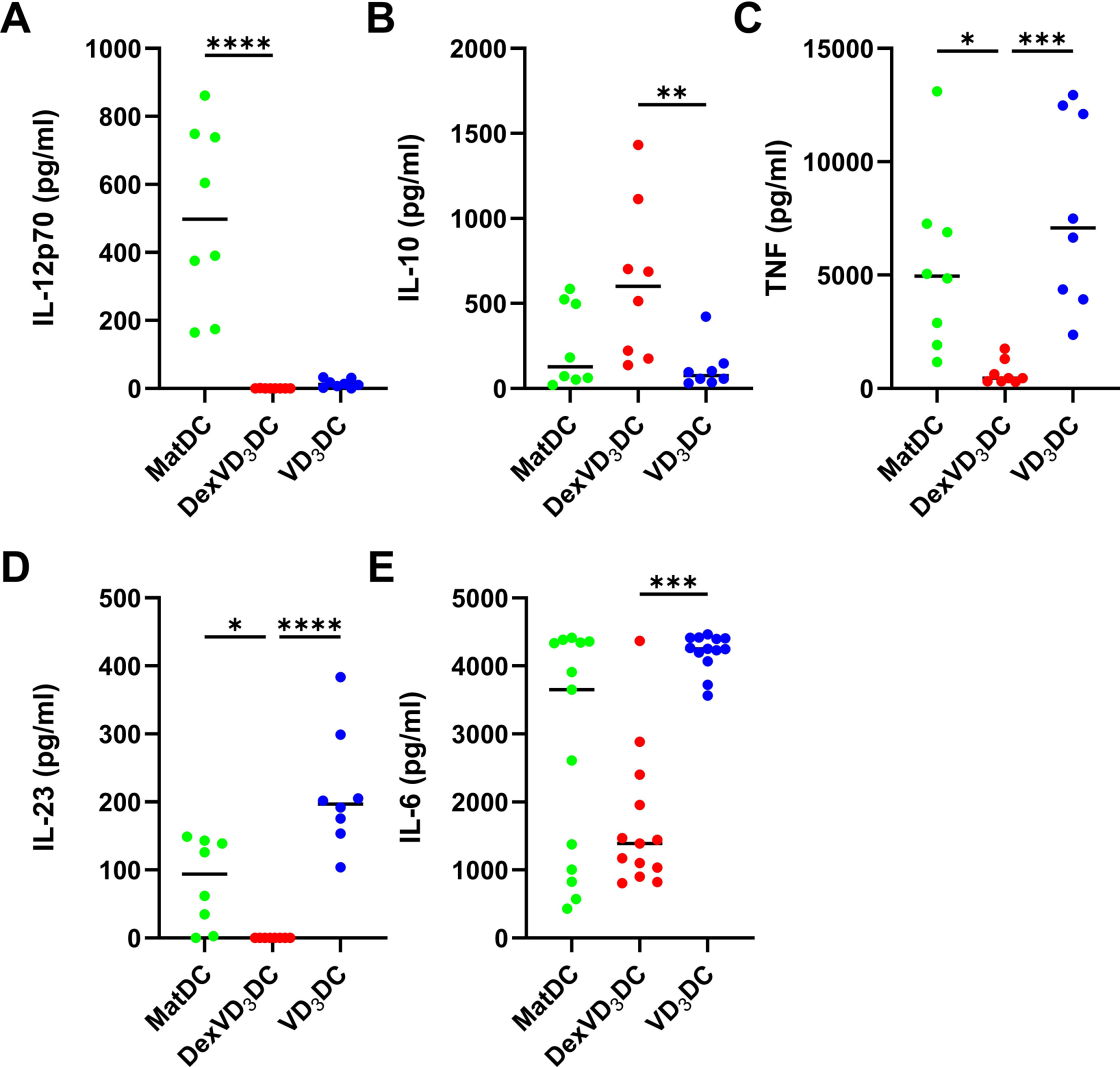
Cytokine secretion of tolDC compared to MatDC controls. Concentration (pg/ml) of **(A)** IL-12p70, **(B)** IL-10, **(C)** TNF, **(D)** IL-23 and **(E)** IL-6 in culture supernatants of MatDC (green), DexVD3DC (red) and VD3DC (blue) was measured using either Luminex® technology **(A–D)** or the V-plex Plus Human Proinflammatory Panel 1 Kit (Meso Scale Discovery) **(E)**. Data shown as individual values where n = 8 **(A–D)** or n = 13 **(E)** with a horizontal line representing the median value. Statistical significance was determined by performing Kruskal-Wallis and Dunn’s multiple comparisons tests. Significance is represented as *p < 0.05, **p < 0.01, ***p < 0.001 and ****p < 0.0001.

The function of different DC subtypes is linked to the profile of molecules expressed at the cell surface. Aligning to protocols for tolDC generation in research and clinical trial studies (1–4), we compared the phenotypes of tolDC generated with vitamin D_3_ alone (VD_3_DC) and in combination with dexamethasone (DexVD_3_DC) by measuring expression of a set of DC maturation and regulatory -associated markers using flow cytometry (**Figure 1**). Characteristic markers of DC maturation (CD83, CD86, HLA-DR) were lowly expressed in immature DC (ImDC) and increased upon maturation with LPS (MatDC). Both DexVD_3_DC and VD_3_DC maintained low levels of CD83 upon LPS maturation with intermediate expression of CD86, whilst HLA-DR was highly expressed by DexVD_3_DC but not by VD_3_DC. In terms of expression of regulatory cell surface molecules, a different pattern was observed between the two tolDC types. DexVD_3_DC but not VD_3_DC showed a significant reduction in expression of programmed-death ligand 1 (PD-L1) compared to MatDC. Furthermore, DexVD_3_DC uniquely showed high expression of the inflammation resolution molecule MERTK, whilst VD_3_DC expressed the highest level of the inhibitory receptor ILT3. Differences were also found for the cytokine secretion profiles of the two tolDC types (**Figure 2**). DexVD_3_DC produced significantly lower levels of pro-inflammatory IL-6, IL-12p70, IL-23 and TNF, and higher levels of anti-inflammatory IL-10 compared to MatDC. Interestingly, although VD_3_DC also showed lower levels of IL-12p70 production compared to MatDC, they did not produce high levels of IL-10 after LPS stimulation but produced high levels of TNF. These data show that the two tolerogenic treatments have contrasting effects on the resulting tolDC in terms of the expression of regulatory molecules expressed or secreted, suggesting that these cells may exert their tolerogenic effect through different mechanisms.

### DexVD_3_DC and VD_3_DC exert differential effects on CD4^+^ T cell proliferation and IFN-γ production

**Figure 3:**
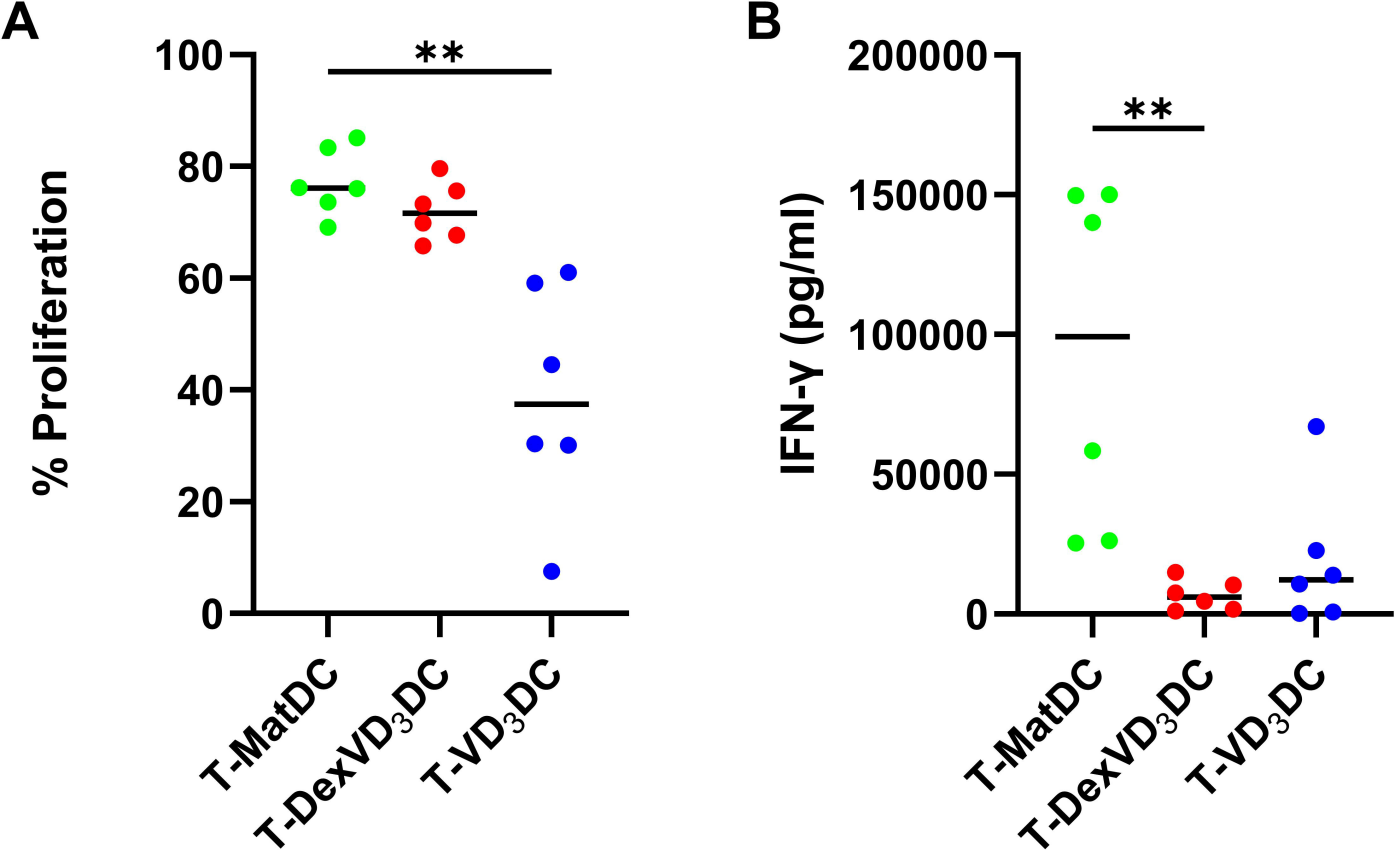
Effect of tolDC on the CD4+ T cell response compared to MatDC controls. **(A)** Percentage proliferation of the CD4+ T cells was measured using flow cytometry analysis of the CellTrace™ proliferation tracking dye and **(B)** concentration of IFN-γ (pg/ml) in supernatants was determined using the V-plex Plus human proinflammatory panel 1 kit (Meso Scale Discovery). Data shown as individual values where n = 6 with a horizontal line representing the median value. Statistical significance was determined by performing Kruskal-Wallis and Dunn’s multiple comparisons tests. Significance is represented as **p < 0.01.

The T cell stimulatory capacity of tolDC types was determined by co-culture with allogeneic naïve T cells obtained from healthy donors (**Figure 3**). VD_3_DC-primed T cells (T-VD_3_DC) showed reduced proliferation compared to T-MatDC, whereas priming of T cells with DexVD_3_DC (T-DexVD_3_DC) yielded comparably high levels of proliferation to T cells primed with MatDC (T-MatDC) (**Figure 3A**). Both T-VD_3_DC and T-DexVD_3_DC, however, showed a significantly reduced production of pro-inflammatory IFN-γ compared to T-MatDC, suggesting that both tolDC types exerted an immunomodulatory response (**Figure 3B**).

### Transcriptomic characterisation of tolDC primed-CD4^+^ T cells from RA patients

To compare the functional implications of different tolDC types in-depth and in a disease-relevant setting, we investigated their effect on the gene expression profile of CD4^+^ T cells that had been primed in co-culture with allogeneic DexVD_3_DC, VD_3_DC or MatDC. We established allogeneic DC/PBMC co-cultures with PBMC from four treatment-naïve RA patients and after 6 days of culture we extracted the CD4^+^ T cells that had expanded by fluorescence-activated cell sorting (FACS). NanoString technology was used to analyse expression of 785 genes involved in immune activation, immune suppression, immune status, immune checkpoints, epigenetics, metabolism and microenvironment.

**Figure 4:**
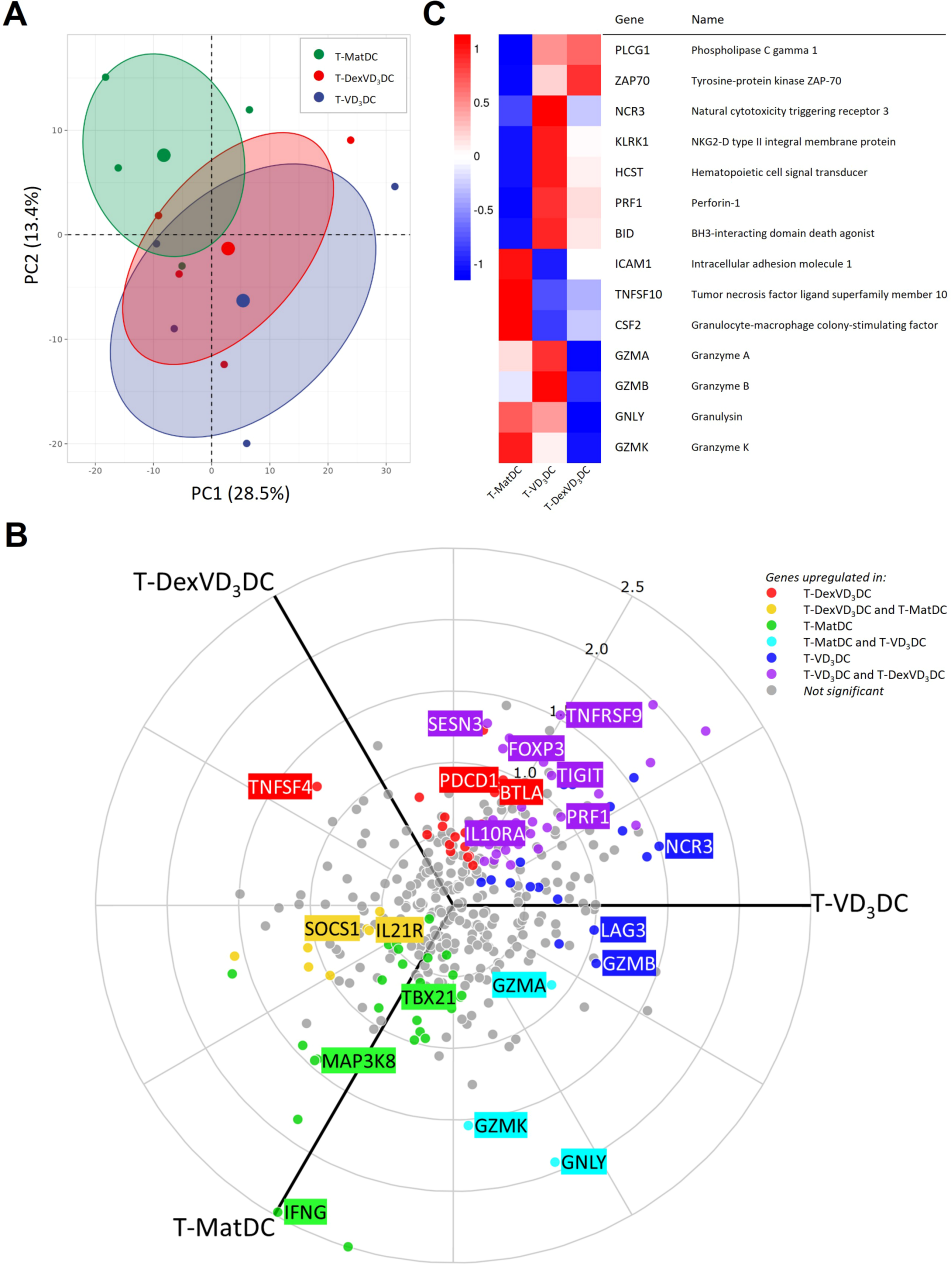
Differential gene expression analysis of CD4+ T cells primed with DexVD3DC, VD3DC or MatDC. CellTrace™ proliferation tracking dye-labelled PBMC from RA patients were cultured with DexVD3DC, VD3DC or MatDC for 6 days. CellTrace™ negative CD4+ T cells were isolated by Fluorescence-activated cell sorting (FACS). Cells were then lysed and gene expression was determined using the NanoString nCounter® platform. **(A)** Principal component analysis (PCA) showing clustering of the different groups. PC1: principal component 1; PC2 principal component 2. Each smaller circle represents an individual sample, coloured as follows: T-MatDC (Green), T-DexVD3DC (Red) and T-VD3DC (Blue). The larger circle in the centre of each ellipses represents the mean value of each group and ellipses represent a 95% confidence interval. **(B)** 3-way comparison polar plot of differentially expressed genes (Benjamini-Hochberg adjusted p value <0.05) between groups. Genes that were significantly upregulated in one group compared to the other two were coloured as follows: T-MatDC (Green), T-DexVD3DC (Red) and T-VD3DC (Blue). Genes significantly upregulated in two groups compared to the reference group are coloured as follows: genes upregulated in T-MatDC and T-VD3DC compared to T-DexVD3DC are cyan, genes upregulated in T-MatDC and T-DexVD3DC compared to T-VD3DC are yellow, and genes upregulated in T-VD3DC and T-DexVD3DC compared to T-MatDC are purple. Non-significant genes (Benjamini-Hochberg adjusted p value > 0.05) are coloured grey. The x, y position of each gene denotes the degree in which it is associated with one or more groups. **(C)** Heatmap of cytotoxicity-related gene expression levels that were significantly differentially expressed (p < 0.05) between T-VD3DC and T-DexVD3DC. N = 4.

Initial principal component analysis (PCA) revealed clustering of tolDC-primed T cells (T-DexVD_3_DC and T-VD_3_DC) and clear separation from T-MatDC at the mRNA level (**Figure 4A**). We identified 113 differentially expressed genes (DEGs) between T-DexVD_3_DC, T-VD_3_DC and T-MatDC (**Supplementary Table 1**). The data revealed upregulation of several key regulatory T cell-specific genes including *FOXP3*, *IL10RA*, *TIGIT*, *SESN3* and *TNFRSF9*, and downregulation of T cell stimulation-linked genes *IFNG* and *MAP3K8* and Th1 transcription factor *TBX21* in T cells co-cultured with both tolDC types compared to the T-MatDC (**Figure 4B**). Transcription factor inference analysis further indicated a profile of reduced transcription factor activity in tolDC-primed T cells when compared to those primed with MatDC, except for FOXO3, which showed increased activity in T-DexVD_3_DC only (**Supplementary Table 2**). Additionally, *LAG3* was upregulated in T-VD_3_DC, whilst *SOCS1* and *IL21R* were downregulated compared to T-DexVD_3_DC and T-MatDC, and *BTLA*, *TNFSF4* and *PDCD1* were upregulated in T-DexVD_3_DC compared to T-VD_3_DC and T-MatDC, once again highlighting distinct regulatory signatures in T cells primed by tolDC generated by different protocols. Gene set enrichment analysis (GSEA) showed chemokine signalling as the pathway with the greatest significance between groups (**Supplementary Table 3**), with expression of *CXCR6*, *CCR2*, *CXCR3*, *CCL2*, *XCL1/2* and *CCL5* increased and *CCL3/L1* and *CXCL3* decreased in T cells co-cultured with tolDC compared to T-MatDC. Indeed, *CXCR6* and *CCR2* were very highly expressed in T-VD_3_DC when compared with T-DexVD_3_DC and T-MatDC, suggestive of a more inflammatory phenotype induced by VD_3_DC (**Supplementary Table 2**). Interestingly, GSEA additionally revealed cytotoxic activity as one of the key pathways with the largest effect size between groups, showing that 9 cytotoxicity-related genes were differentially expressed (**Supplementary Tables 1**, **3**). Of these, *GZMA*, *GNLY*, *GZMK* were upregulated in T-MatDC and T-VD_3_DC, *NCR3* and *GZMB* were upregulated in T-VD_3_DC only, and *PRF1* was upregulated in T-DexVD_3_DC but to a greater extent in T-VD_3_DC (**Figure 4C**). A signature of 11 transcription factors was inferred with reduced activity in T-VD_3_DC when compared to T-DexVD_3_DC, of which only TFAP2A was predicted to regulate GZMB expression. Transcription factors JUNB and HLX were inferred to have significantly increased activity in T-VD_3_DC. Transcription factors known to activate cytotoxicity genes (e.g., EOMES and TBX21) were among those with reduced activity in both T-tolDC when compared to T-MatDC, indicating a complex and non-canonical induction of cytotoxicity by VD_3_DC that requires future exploration (**Supplementary Table 2**). Notably, the relative expression of cytotoxicity associated genes was the lowest in T-DexVD_3_DC, potentially revealing a major difference in the regulatory activities of DexVD_3_DC and VD_3_DC in addition to indicating a possible role for dexamethasone – treated DC in suppressing cytotoxic gene expression in T cells.

### VD_3_DC, but not DexVD_3_DC, induce granzyme B expression and cytotoxic activity in naïve CD4^+^ T cells

**Figure 5:**
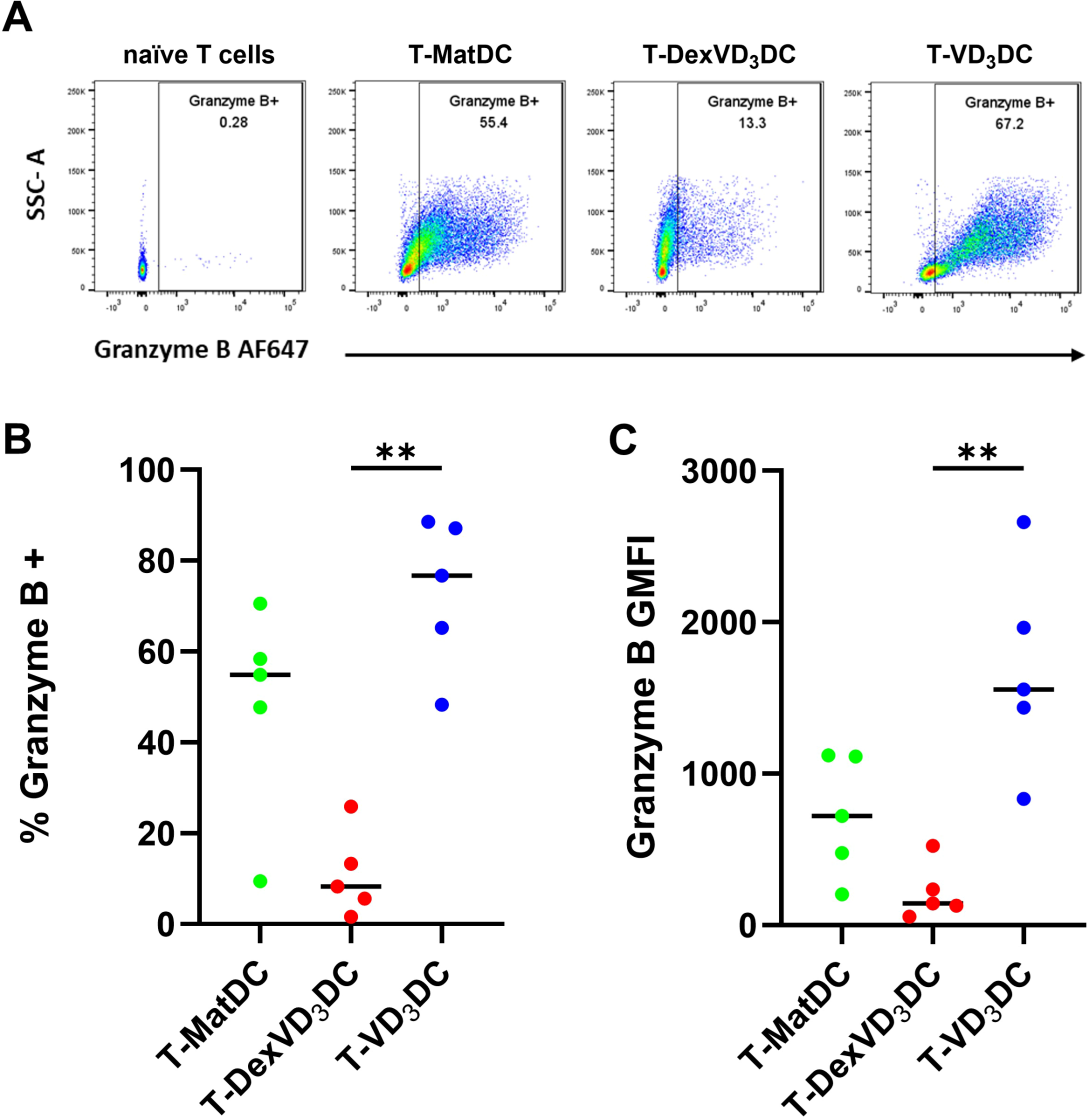
Analysis of intracellular granzyme B expression in DC-primed T cells by flow cytometry. Naïve healthy donor CD4+ T cells were co-cultured with allogeneic MatDC (green), DexVD3DC (red) or VD3DC (blue) for 6 days. **(A)** One representative experiment of granzyme B expression in live naïve T cells, T-MatDC, T-DexVD3DC and T-VD3DC. **(B)** Percentage positive of live cells, **(C)** Geometric Mean Fluorescence Intensity (GMFI) of granzyme B Data shown as individual values where n = 5 with a horizontal line representing the median value. Statistical significance was determined by performing Kruskal-Wallis and Dunn’s multiple comparisons tests. Significance is represented as **p<0.01.

As an indicative marker and key component of the cytotoxic killing apparatus which was markedly differentially expressed at the gene expression level, we looked to confirm the expression of granzyme B at the protein level in CD4^+^ T cells co-cultured with the different DC types. To this aim, we used naïve CD4^+^ T cells from healthy donors, which lack expression of granzyme B (**Figure 5A**), thus enabling us to investigate the induction of this protein. In alignment with the mRNA data (**Figure 4C**), there was a notable and significant difference in the expression of granzyme B protein in T cells that had been primed by VD_3_DC as compared to DexVD_3_DC, with VD_3_DC inducing a higher proportion of cells expressing this protein, as well as a higher level of expression in those cells that expressed granzyme B (**Figures 5B, C**).

**Figure 6:**
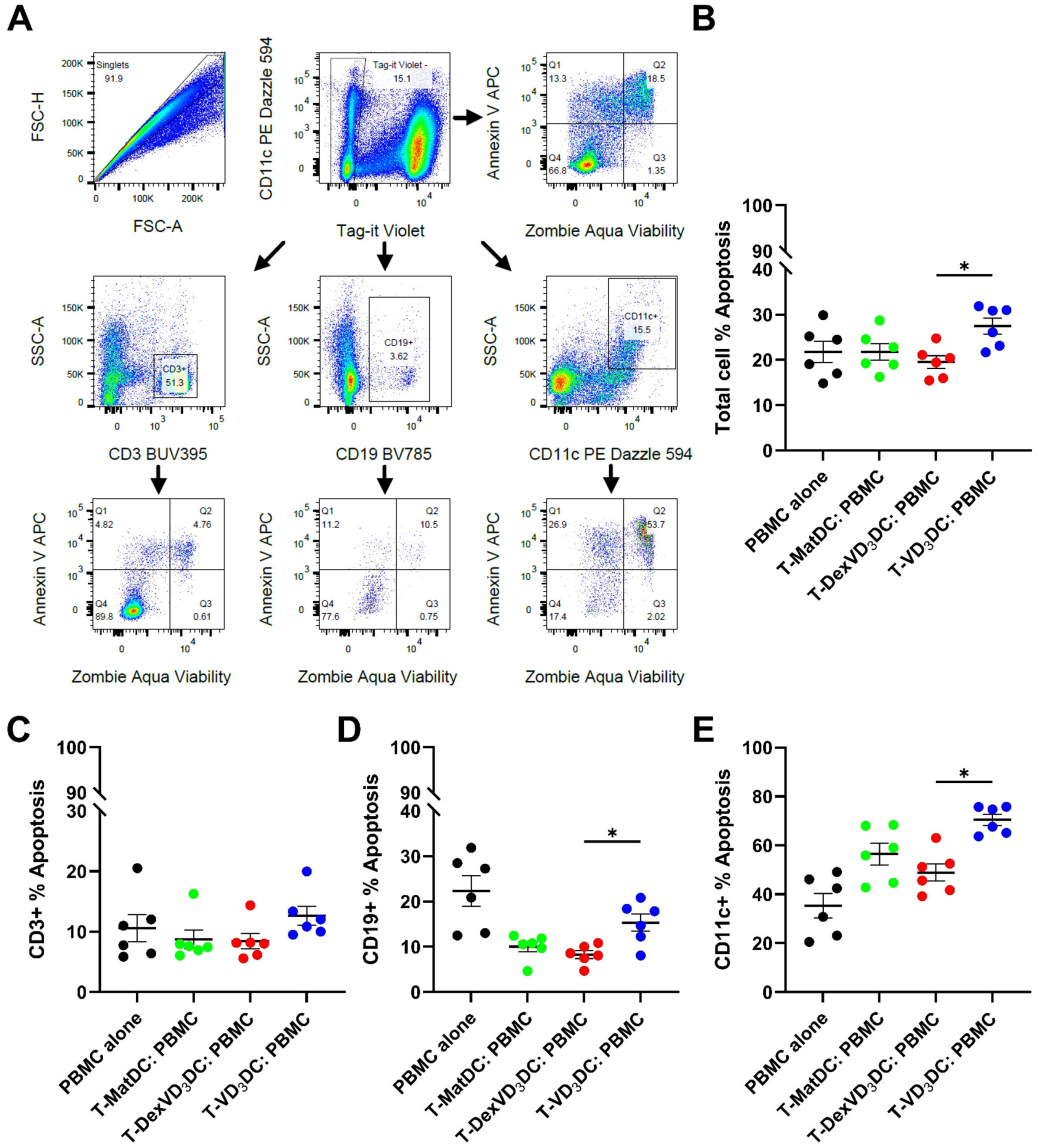
Assessment of functional cytotoxicity of DC-primed T cells. MoDC (1x104/well) were co-cultured with allogeneic naïve T cells at a 1:10 DC: T cell ratio for 6 days. On day 6, cells were labelled with Tag-it Violet™(Biolegend) to differentiate them from the target PBMC and 1x105/well were co-cultured with thawed PBMC from the original moDC donor at a 2:1 T cell:PBMC ratio for 24 hours. **(A)** Example gating strategy used to determine percentage of apoptosis in **(B)** total PBMC, **(C)** CD3+ cells, **(D)** CD19+ cells and **(E)** CD11c+ cells. Doublets and cells that were stained positive for Tag-it Violet™ were excluded. Apoptosis was then determined either in the total PBMC population (A top right) or in the individual populations specified using Zombie Aqua™(ZA) and Annexin V (AV) (Biolegend). Cells that were AV+ (Q1:ZA-, AV+ and Q2: ZA+, AV+) were considered apoptotic. Data shown as individual values with mean ± SEM where n = 6. Statistical significance was determined by performing a one-way ANOVA with Tukey’s paired *post hoc* analysis on ln-transformed data. Significance is represented as *p<0.05.

To determine whether the increased expression of granzyme B in T-VD_3_DC translated to a functional increase in cytotoxicity, we co-cultured T-VD_3_DC, T-DexVD_3_DC and T-MatDC with PBMC derived from the allogeneic donor from which the DC types were generated (to facilitate secondary activation of the primed T cells via the same allogeneic HLA) for 24 hours and measured the percentage of cells undergoing apoptosis. Apoptosis was determined using the gating strategy shown (**Figure 6A**). Indeed, co-culture with T-VD_3_DC resulted in an increased percentage of apoptotic PBMC compared to co-culture with T-DexVD_3_DC (**Figure 6B**). In addition, we determined which cell types were most susceptible to killing by T-VD_3_DC. Our results revealed T-VD_3_DC induced a higher percentage of apoptosis of CD11c^+^ cells compared to T-DexVD_3_DC, but not CD3^+^ cells. We noted that, compared to the PBMC-only control group, CD19^+^ cells showed reduced apoptosis when co-cultured with any of the allogeneic DC-activated T cell types, indicative that survival signals (T cell help) may partially offset cytotoxic CD4^+^ T cell activity. However, apoptosis was significantly higher in CD19^+^ cells cultured with T-VD_3_DC compared to T-DexVD_3_DC, thus suggesting that B cells and myeloid cells, but not T cells are the preferential target of cytotoxic killing in our model (**Figures 6C–E**). Overall, these data indicate VD_3_DC are inducing a cytotoxic phenotype in CD4^+^ T cells at the mRNA and protein level, which translates to an increase in functional cytotoxicity in these cells.

## Discussion

This study identified functional differences between DexVD_3_DC and VD_3_DC, indicating divergent regulatory activity between these tolDC cell types, and highlighting non-canonical induction of cytotoxic CD4^+^ T cells.

Comparison of the phenotype and the T cell stimulatory capacity of these tolDC types showed that they conformed to the cell characteristics previously reported by us and others (8, 10, 19, 26, 33). Both DexVD_3_DC and VD_3_DC displayed a semi-mature phenotype and expressed their typical regulatory cell surface markers. Thus, DexVD_3_DC expressed the inflammation resolution marker MerTK, which is upregulated by dexamethasone in monocyte-derived DC (34, 35) and is currently being used as a quality control marker for clinical-grade DexVD_3_DC in our AuToDeCRA-2 trial. VD_3_DC, on the other hand, did not express high levels of MerTK but did display a marked increase in the regulatory markers PD-L1 and ILT3, which have been implicated in the induction of Tregs and T cell anergy (36, 37). We also noted significant differences in the cytokine secretion profiles of these tolDC types (in response to LPS), with DexVD_3_DC being superior in terms of IL-10 production, and VD_3_DC (but not DexVD_3_DC) producing IL-6, IL-23 and TNF levels similar to mature DC. Whilst IL-10 undoubtedly plays an essential role in controlling pathological inflammation and the induction of Tr1 cells (38, 39), TNF can exert both pro-inflammatory and anti-inflammatory roles, depending on the context. For example, TNF acts synergistically with IL-1β to drive the maturation of immunogenic DC (40, 41), but it can also promote the expansion and function of FoxP3^+^ Tregs through TNFR2 signaling (42). One cytokine feature that was highly similar between the two tolDC types was their very low or undetectable production of IL-12p70, likely explaining their poor ability to induce IFN-γ production in allogeneic naïve CD4^+^ T cells. The observation that DexVD_3_DC have a relatively strong ability to induce T cell proliferation but not IFN-γ secretion has been noted by us previously in both allogeneic and autologous settings (8, 12). It is probable that our proliferation findings are linked to the pronounced differences in HLA-DR expression level between tolDC types, whereby DexVD_3_DC express HLA-DR levels equivalent to matDC and VD_3_DC express HLA-DR levels equivalent to immature DC. HLA expression has implications for signal avidity through the TCR, both in our allogeneic experiments and in *in vivo* antigen specific systems. HLA is likely to be an important factor in determining downstream T cell effector functions in a therapeutic context. Taken together, these results showed some similarities but also clear differences in phenotypic and functional characteristics of these two tolDC types.

It should be noted that variations in tolDC phenotype may not only depend on the pro-tolerogenic agents used, but also on the patient population from which the tolDC have been generated. For example, elegant work (43) has shown that VD_3_DC from MS patients exhibited more pro-inflammatory features than VD_3_DC from healthy donors, due to alterations in the aryl hydrocarbon receptor (AhR) and NF-κB pathways, impairing their tolerogenic function. In contrast, this issue does not seem to affect DexVD_3_DC to the same extent, at least not when generated from the peripheral blood of RA patients, which are highly similar to DexVD_3_DC from healthy donors in terms of their cell surface phenotype, cytokine production profile and T cell suppressive action (10). It could be speculated that Dex’s broad and potent immunosuppressive actions are less sensitive than VD3 to the pro-inflammatory milieu from which the monocytes were obtained. Nevertheless, patient background should be considered when designing tolDC therapy. In the case of the impaired tolerogenic function of VD_3_DC from MS patients (43), co-treatment with the drug dimethyl fumarate to enhance the therapeutic action of VD_3_DC was proposed.

Because these drug-induced tolDC have been developed for therapeutic application in autoimmune conditions, we investigated their modulatory effects on CD4^+^ T cells derived from four drug-naïve early RA patients. Although the small size of this cohort constitutes a limitation of this study, its strength lies in the fact that the use of these patient samples precluded interference from disease-modifying anti-rheumatic drugs. We found similarities and differences in the regulatory gene profile induced by these tolDC. Most notably, we show a prominent signature of FOXO3 transcriptional regulation in T cells primed by DexVD_3_DC and not in VD_3_DC which may govern differences in regulatory phenotype and functions (44). We also show *LAG3*, a regulatory gene that we had previously reported to be upregulated in T cells primed by DexVD_3_DC (12), was a more prominent feature of T cells primed by VD_3_DC. This apparent discrepancy is most likely explained by the different comparator groups in our previous study, where we compared the T cell priming effects of DexVD_3_DC to those of immature DC. Other differences between the two studies are the mode of T cell activation (allogeneic versus antigen-specific) and the donors (RA patients versus healthy controls). Nevertheless, the current study confirms that both tolDC types induce the expression of regulatory genes in CD4^+^ T cells from RA patients, but the pattern of expression differs and the continued exploration of tolDC responses in different contexts is both necessary and valuable.

Interestingly, our analyses revealed that VD_3_DC were superior at inducing a cytotoxic gene signature in CD4^+^ T cells that is usually considered characteristic of CD8^+^ T cells. Indeed, VD_3_DC induced expression of granzyme B protein, which discriminated most highly between the T-DexVD_3_DC and T-VD_3_DC cells at the transcriptional level. In line with our observed granzyme B expression, VD_3_DC-primed T cells exhibited the highest cytotoxic activity, with their killing mainly directed at myeloid cells and B cells. Rather than being clearly linked with activity of transcription factors previously described to activate cytotoxicity genes such as Eomes (45) Blimp-1, T-bet (46), and Runx3 (47), the superior induction of T cell cytotoxicity by VD3DC appears to be complex. Our findings suggest that transcription factors including JUNB and HLX may influence granzyme expression and cytotoxic function through their role in T-helper cell lineage commitment (48).

The roles of individual components of cytotoxicity apparatus such as granzyme B are complex (49). In addition to its canonical, perforin-dependent role in NK and CD8^+^ T cell cytotoxicity, granzyme B is known to have extracellular activity (50). It has been identified in RA serum, plasma and synovial fluid and is thought to be associated with inflammation and degradation of cartilage proteoglycan (51–53). Conversely, granzyme B has complex perforin-independent and receptor-mediated functionalities and its expression in B cells and plasmacytoid DC has been associated with T cell regulation in inflammatory diseases and cancer (54, 55). The wide-ranging activities of cytotoxicity-related proteins induced in T cells by VD_3_DC may be favourable or detrimental in disease settings, and further exploration is required to better understand the implications of this novel function of tolDC.

Despite key commonalities such as identification of a prominent role for JunB in regulatory function of T-VD_3_DC, our data are to some extent in contrast to a recent study by Navarro-Barriuso et al. (56), who showed that VD_3_DC slightly reduced the secretion of granzyme B in autologous CD4^+^ T cells in an antigen-specific setting. Because they used a recall antigen (tetanus toxin), the responding T cells will have been memory rather than naïve, which may explain this contrast. Another possible explanation is the different protocols used for the generation of VD_3_DC, with the dose of VD_3_ (1 vs 10 nM) and the mode of DC maturation (cytokine cocktail vs LPS) used as the most likely considerations. As VD_3_DC are being explored and trialled as a therapeutic for the treatment of autoimmune disease, it will be important to address the basis of these contrasting findings in future studies.

Our observation that VD_3_DC-induced cytotoxic CD4^+^ T cells were able to kill antigen-presenting cells (APC) including B cells may be interpreted as a mechanism by which these cells could exert therapeutic effects in autoimmune conditions, as the killing of professional APC that present autoantigen is likely to lead to a reduction in the activation of autoantigen-specific T cells. Furthermore, we show that while CD19^+^ B cell survival is supported by the presence of T cells, T-VD_3_DC uniquely subvert this T cell help to induce significant B cell death. Our findings are of interest in the context of the antibody-dependent and independent roles of B cells, which are already key targets for therapeutic intervention in autoimmune conditions. Indeed, cytotoxic function has been described as a feature of certain Tregs (57). However, it should be noted that the large majority of granzyme B-expressing CD4^+^ T cells did not express FoxP3 protein (data not shown), indicating that these cells are not necessarily ‘classical’ Tregs. Whether they are more representative of induced Tr1 cells or terminally differentiated effector cytotoxic CD4^+^ T cells will require further investigation through extensive single-cell profiling by scRNAseq and/or multiparametric flow cytometry methods. In addition, such investigations may also shed further light on the apparent heterogeneity of the VD3DC-primed CD4^+^ T cells.

The potent cytotoxic activity of these VD_3_DC-primed CD4^+^ T cells is a striking feature, which is unlikely to be a consequence of the allogeneic *in vitro* stimulation of these cells. It has previously been shown that induction of cytotoxicity in CD4^+^ T cells upon allogeneic stimulation occurs after long-term *in vitro* culture (58, 59), but not short-term culture (58). The comparison between T cells primed with the different DC types under identical short-term *in vitro* culture conditions supports the notion that the enhanced induction of cytotoxic T cells by VD_3_DC does reflect a true mechanism by which these cells act. Moreover, VD_3_DC express significantly lower levels of HLA-DR (**Figure 1**) and have impaired capacity to induce allogeneic T cell proliferation (**Figure 3**), thus making it highly unlikely that cytotoxicity was caused by artifactual overstimulation of T cells. In this context it is interesting to note that low doses of antigen tend to drive more potent cytolytic activity in CD4^+^ T cells (60), thus aligning with our observation that VD_3_DC, with the lowest potency in terms of inducing allogeneic MHCII-dependent T cell activation, displayed the strongest ability to induce cytotoxicity. While the same study demonstrated that inflammatory cytokines were not required for cytolytic activity of CD4^+^ T cells (60), the induction of cytotoxicity by DC does correlate with expression of IL-6, IL-23 and TNF in our experiment. These cytokines are potently inhibited by dexamethasone (8, 61) and are not positively associated with inducing cytotoxicity in T cell literature to date. As IL-6 is a hallmark of tolerogenic VD_3_DC (62), a possible mechanistic role in supporting a cytotoxic T cell phenotype requires further investigation.

It is now accepted that cytotoxic CD4^+^ T cells are not simply an *in vitro* phenomenon. Expansion of cytotoxic CD4^+^ T cells has been observed in patients with chronic viral infections, and these cells are now considered to play a critical role in anti-viral defence through the killing of virus-infected MHCII-expressing cells (63–66). However, they have also been implicated in the pathogenesis of autoimmune diseases, with expansion of these cells being positively correlated with disease severity and progression in MS patients (67) and driving excess cardiovascular mortality in RA patients (68). In addition to the expression of granzyme B alongside other cytotoxic molecules, these CD4^+^ T cells produce high levels of IFN-γ and IL-17, accumulate in inflammatory autoimmune lesions, are autoreactive and have cytolytic capacity towards target tissues; for example, in MS they have been shown to kill oligodendrocytes (69–73). It should be noted that the high expression of granzyme B in VD_3_DC-primed CD4^+^ T cells was not accompanied by high levels of secreted IFN-γ in our study; on the contrary, levels of this cytokine were significantly reduced as compared to MatDC-primed T cells. Moreover, research by other groups has shown that VD_3_DC polarise T cells towards high IL-10 and low IFN-γ and IL-17 cytokine production (20), thus it could be speculated that the cytotoxic activity observed in these cells is more related to their regulatory, rather than pathogenic, function. Nonetheless, further work is needed to establish whether autoantigen-loaded VD_3_DC induce cytotoxic autoantigen-specific CD4^+^ T cells *in vivo* and if so, whether the beneficial effects these cells may have through the killing of MHCII^+^ professional APC, autoantibody-producing B cells and possibly other resident cells such as fibroblasts, outweigh any potential pathogenic activity.

In conclusion, our data show considerable divergence between the regulatory effects of DexD_3_DC and VD_3_DC, with only the latter displaying potent ability to induce cytotoxic CD4^+^ T cells. Further work is needed to establish to what extent the induction of cytotoxic CD4^+^ T cells by VD_3_DC takes place *in vivo*, and whether it contributes to the therapeutic action of these tolDC.

## Data Availability

The original contributions presented in the study are publicly available. This data can be found here: NCBI’s Gene Expression Omnibus https://www.ncbi.nlm.nih.gov/geo/query/acc.cgi?acc=GSE313751.

## Supplementary material

Supplementary Data Sheet 1 Raw count data for all genes across all samples. Data was acquired on the NanoString nCounter® platform using the Immune Exhaustion panel (NanoString Technologies, Seattle, WA, USA). Includes gene name and accession number.

Supplementary Table 1 All differentially expressed genes in CD4+ T cells primed with DexVD3DC, VD3DC or MatDC after normalisation and quality control steps. CellTrace™ proliferation tracking dye-labelled PBMC from RA patients were cultured with DexVD3DC, VD3DC or MatDC for 6 days. CellTrace™ negative CD4+ T cells were isolated by Fluorescence-activated cell sorting (FACS). Cells were then lysed and gene expression was determined using the NanoString nCounter® platform. A negative binomial generalised linear model (Bioconductor package DESeq2) was fitted to the raw count data following normalisation and quality control. A likelihood ratio test was performed to compare variation between groups, followed by pairwise comparisons, using Wald tests, between T-MatDC, T-DexVD3DC and T-VD3DC. Genes with a raw p-value < 0.05 and a Benjamini-Hochberg adjusted p-value < 0.05 for the LRT and Wald test, respectively, were considered to be differentially expressed.

Supplementary Table 2 Transcription Factor (TF) inference analysis results. TF activities were inferred using the Univariate Linear Model (ULM) method within the decoupleR package using the gene-level Wald statistics and the human CollecTRI regulatory network as input. Signed TF-Gene interaction weights from CollectTRI were regressed against the differential expression statistics to compute activity scores per TF.

Supplementary Table 3 Gene Set Enrichment Analysis (GSEA) results. GSEA was conducted using the Fast Gene Set Enrichment Analysis (FGSEA) method outlined by Korotkevich et al. (Korotkevich et al., 2021) (31). Gene sets were defined based on the NanoString Immune Exhaustion panel annotations and were considered significant if they had a p-value < 0.05.
